# Prevalence of celiac disease in patients with Down syndrome: a meta-analysis

**DOI:** 10.18632/oncotarget.23624

**Published:** 2017-12-23

**Authors:** Yang Du, Ling-Fei Shan, Zong-Ze Cao, Jin-Chao Feng, Yong Cheng

**Affiliations:** ^1^ Center on Translational Neuroscience, College of Life and Environmental Sciences, Minzu University of China, Beijing 100081, China

**Keywords:** celiac disease, Down syndrome, prevalence, meta-analysis

## Abstract

**Background:**

The association between Down syndrome and celiac disease has been reported by many studies. However, the prevalence of celiac disease (CD) in Down syndrome (DS) varies considerably across studies (from 0 % to 19 %). The aim of this study was to use meta-analysis to exam the prevalence of CD in patients with DS.

**Methods:**

A systematic search of English articles from Pubmed, Web of Science and CNKI without year limitation. Data were extracted by two independent observers and pooled using a random effects model by the Comprehensive Meta-Analysis Version 2 software.

**Results:**

A pooled analysis, based on 31 studies included 4383 individuals, revealed prevalence of biopsy-confirmed CD of 5.8 % (95 % CI = 4.7-7.2 %) in patients with DS. Sub-group analysis showed a slightly higher prevalence of CD in children with DS (6.6 %; 17 studies), than in age mixed samples with both children and adults (5.1 %; 13 studies). In addition, most of the studies included in this meta-analysis were from Europe and America, with the prevalence of celiac disease of 6 % (21 studies) and 5.7 % (6 studies) in DS patients, respectively. Furthermore, meta-regression analysis suggested that proportion of antibody-positive individuals that underwent small intestine biopsy had moderating effect on the outcome of the meta-analysis.

**Conclusions:**

These results demonstrated that patients (children) with Down syndrome had high prevalence of CD (more than one in twenty). The prevalence is high enough to motivate screening CD in DS children.

## INTRODUCTION

Celiac disease is an autoimmune disorder which affects people who are genetically disposed to it [1]. The disease is characterized by villous atrophy of the small intestine induced by wheat, rye, and barley in the food [2]. Although the prevalence of CD varies between different regions of the world, the average prevalent rate of the disease was reported to be between 0.5 % to 1 % [3, 4].

Compared to the general population, literature has provided evidence that CD is more frequent in patients with some genetic and autoimmunological diseases, these diseases include type 1 diabetes [5, 6], autoimmune thyroid disease [7, 8], autoimmune hepatitis [9] and Down syndrome (DS) [10]. Although the prevalence of CD in those diseases varied substantially among studies, a systematic review with meta-analysis showed that 6 % of patients with type 1 diabetes have biopsy-confirmed celiac disease [11]. In autoimmune thyroid disease, a pooled analysis with 6024 patients found a prevalence of biopsy-verified CD of 1.6 %, and the prevalence of CD was higher in children with autoimmune thyroid disease [12].

Although several studies have demonstrated a high prevalence of CD in patients with DS, both in children and adults, the prevalence of CD in patients with DS has been reported to be varied from 0% to 19% [1, 13–18], this may contribute to the lack of consensus on screening of CD in patients with DS. Therefore, a systematic review and meta-analysis is necessary to address the inconsistent clinical data.

The aim of this study was to examine the prevalence of CD in patients with DS with systematic review and meta-analysis. Sub-group and meta-regression analyses were also used to address the between-study heterogeneity found in this meta-analysis. The meta-analytic technique allows data from individual studies to be pooled quantitatively and improve the strength of the clinical data.

## RESULTS

**Figure 1:**
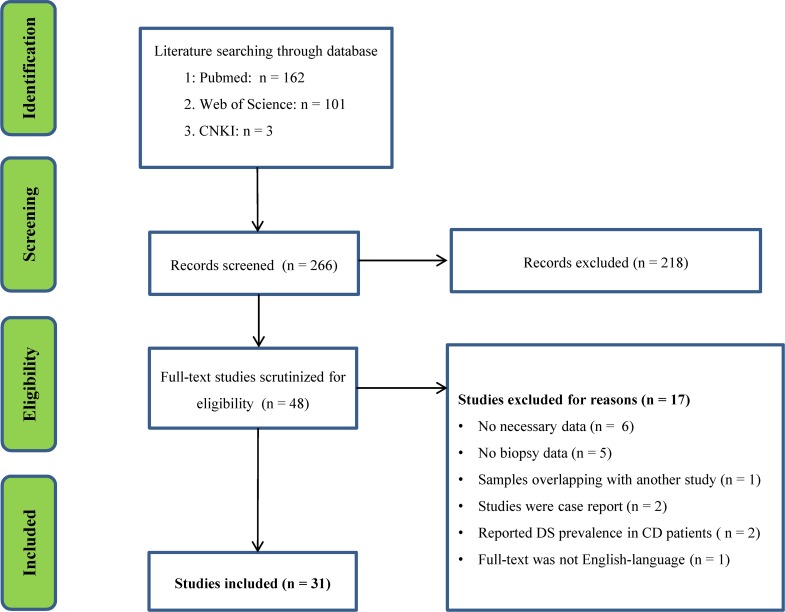
PRISMA flowchart of the literature search

The initial search generated 162 records from PubMed, 101 records from Web of Science and 3 records from CNKI. Screening titles and abstracts resulted in identification of 48 papers for full text scrutiny. After reading the full text of the 48 articles, we excluded 17 studies for the following reasons: lacked necessary data (six studies); lacked biopsy data (five studies); studies were case reports (two studies); reported DS prevalence in CD patients (two studies); full text was not English-language (one study); samples were overlapping with another study (one study). Therefore, a total of 31 studies assessing CD prevalence in DS patients were included in this meta-analysis [1, 10, 13–41] (Flowchart see Figure 1).

### Main association of CD with DS

**Figure 2:**
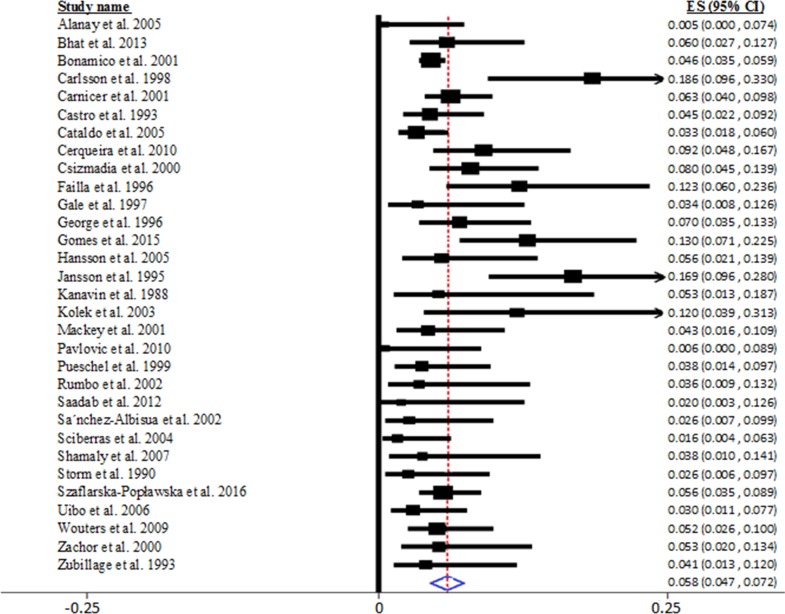
Pooled prevalence of biopsy-verified celiac disease in patients with Down syndrome

Random-effects meta-analysis suggested that the pooled prevalence of CD in DS patients was 5.8% (95% CI = 4.7-7.2 %), extracted from 31 studies encompassing 4383 patients with DS, as shown in Figure 2. However, we noted significant heterogeneity between studies in this meta-analysis (Q_30_ = 37.544, I^2^ = 54.426, P < 0.001).

### Sub-group analysis

To investigate the potential sources that explained the heterogeneity found in this meta-analysis, we first performed sub-group analysis considering age at the CD testing.

17 studies in this meta-analysis analyzed CD prevalence in children with DS, the other 13 studies analyzed CD prevalence both in children and adults with DS, and one study analyzed CD prevalence in adults with DS. Therefore, we performed sub-group analysis based on whether samples were only obtained from children. The meta-analysis found a slightly higher CD prevalence in children with DS (6.6 %; 95% CI: 4.7-9.2 %), than in age mixed samples with both children and adults (5.1 %; 95% CI: 4.0-6.4 %). However, the heterogeneity did not reduce for studies assessing DS prevalence in only children with DS (Q_16_ = 37.544, I^2^ = 57.383, P = 0.002), whereas the heterogeneity for studies assessing DS prevalence in both children and adults with DS significantly reduced (Q_12_ = 17.957, I^2^ = 33.175, P = 0.117).

We next carried out sub-group analysis to test whether regional difference had moderating effect on the outcome of the meta-analysis. 21 studies included in this meta-analysis were from Europe, and the pooled data showed a prevalence of 6 % (95 % CI: 4.6-7.8 %). Similarly, the prevalence of CD in DS patients was 5.7 % (95 % CI: 3.4-9.3 %) in America from 6 studies. In addition, significant heterogeneity was found for studies from Europe (Q_20_ = 54.727, I^2^ = 63.455, P < 0.001), whereas no significant heterogeneity was observed for studies from America (Q_5_ = 8.617, I^2^ = 41.977, P =0.125).

### Meta-regression analysis

**Figure 3:**
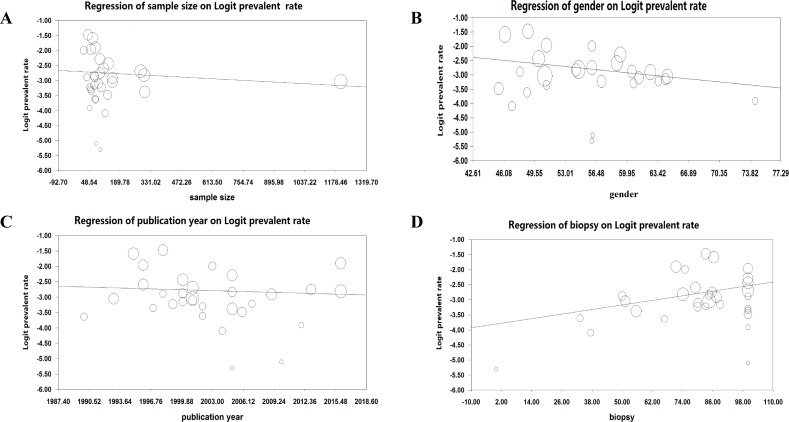
Association between sample size **(A)**, gender **(B)**, publication year **(C)**, proportion of antibody-positive individuals that underwent small intestine biopsy **(D)** and effective size (Logit prevalent rate). The sizes of the circles are proportional to study weight.

Meta-regression analyses were performed to analyze whether the continuous variables affected the observed heterogeneity in this meta-analysis, these variables include sample size, publication year, gender (proportion of male) and proportion of antibody-positive individuals that underwent small intestine biopsy. The results showed that sample size (regression coefficient [SE], −0.0004 [0.0004]; 95% CI, −0.0012 to 0.0004; P = 0.32), publication year (regression coefficient [SE], −0.009 [0.018]; 95% CI, −0.044 to 0.026; P = 0.62) and gender (regression coefficient [SE], −0.031 [0.019]; 95% CI, −0.068 to 0.006; P = 0.11) had no moderating effects on the outcome of the meta-analysis (Figure 3A-3C). However, meta-regression suggested that proportion of antibody-positive individuals that underwent small intestine biopsy positively correlated with the effective size (Figure 3D; regression coefficient [SE], 0.013 [0.006]; 95% CI, 0.0009 to 0.0244; P = 0. 035).

### Sensitivity analysis

When in the sensitivity analysis we omitted a study at a time to assess the influence of an individual study on the pooled prevalence of CD in DS, the prevalence ranged from 5.6 % to 6.0 % after removing an individual study, suggesting that that the pooled prevalence in this meta-analysis was not significantly affected by any single study.

### Publication bias

**Figure 4:**
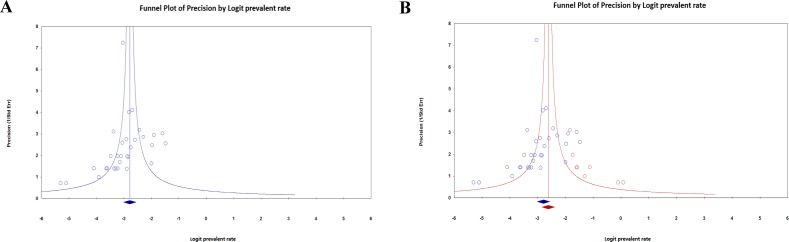
Funnel plot examining publication bias in observed **(A)**, observed and imputed **(B)** studies assessing prevalence of celiac disease in patients with Down syndrome. The plots describe the effective size (Logit prevalent rate) of studies against their precision (inverse of standard error). Blue diamond marker indicates observed pooled effective size, red diamond marker indicates imputed pooled effective size.

Visual inspection the funnel plots suggested presence of publication bias in this meta-analysis (Figure 4A). We used the trim-and-fill procedure to exam the impact of publication bias, and this estimation suggested that eight studies needed to be imputed to generate a symmetric funnel plot. Imputation led to a higher prevalence of CD in patients with DS (Figure 4B), suggesting that the high prevalence of CD in patients with DS observed in this meta-analysis was not caused by publication bias.

## DISCUSSION

To the best of our knowledge, this is the first meta-analysis undertaken to investigate the prevalence of CD in DS patients. In this meta-analysis including more than 4000 children and adults with DS from 31 studies in the literature, 5.8 % DS patients had biopsy verified CD. Through sensitivity analysis, we concluded that no single study significantly influenced the prevalence of CD in DS patients. Although publication bias was found in the studies included in the meta-analysis, results from the trim-and-fill procedure suggested a higher prevalence of CD in DS patients after publication bias has been taken into account. Because the prevalence of CD in DS patients is controversial for more than two decades, due to the inconsistent clinical data from studies, the current study with meta-analytic technique provides strong clinical evidence that at least one in twenty DS patients had CD.

In addition to the 31 studies analyzing prevalence of CD in DS patients, we have identified two studies that assessed the occurrence of DS in patients with CD. A nationwide study from Sweden compared the occurrence of DS in 11749 individuals with biopsy-verified CD between 1973-2008 vs 53887 general population, and concluded that CD was associated with a 6.15-fold increased risk of individuals with DS (95 % CI = 5.09-7.43) [42]. Given that the prevalence of CD in general population has been reported to be about 0.5% to 1% [3, 4], the nationwide study further supported the conclusion in this meta-analysis. Consistently, the other study reviewed 190 patients with CD and found an increased incidence of DS in CD compared to the general population [43].

Although the Celiac Disease Guideline Committee of the North American Society for Pediatric Gastroenterology, Hepatology, and Nutrition recommends CD screening in asymptomatic DS children [44], and the American Academy of Pediatrics recommends testing for CD in DS children with CD-related symptoms [45], there are no guidelines for CD screening in children with DS in other regions or countries. The controversy on CD screening in DS children was largely due to the considerably inconsistent prevalent rate of CD in DS. The significance of the current meta-analysis is that we have included sufficient number of studies with large sample size, and concluded a high prevalence of CD in DS, the finding here therefore should motivate screening for CD in patients with DS, especially in Europe, given that most of the studies included in this meta-analysis were from Europe.

The mechanism underlying the association between DS and CD is unclear. In general, susceptibility to CD is associated with HLA-DQ2 (A1^*^0501-B^*^0201 or A1^*^0201-B^*^0202) and HLA-DQ8 (A1^*^0301-B1^*^0302), and about 95 % and 5% of patients with CD have these haplotypes, respectively [1, 46]. However, it is known that the distribution of HLA genotypes is similar in patients with DS compared to the general population [47], and the immune-related non-HLA loci has been search as candidate genes in DS that leads to the high comorbidity of CD with DS. In addition, studies have demonstrated that patients with DS had increased pro-inflammatory cytokine levels, these cytokines include tumor necrosis factor-α, IL-1β and interferon-gamma [48–50], and the heightened levels of the cytokines may contribute to the occurrence of CD in DS patients. Nevertheless, future studies are necessary to explore the mechanism underlying the association between CD and DS.

This meta-analysis found moderate level of between-study heterogeneity. The strength of this study is that we used sub-group analyses and meta-regressions to address the confounders that explained the heterogeneity. Sub-group analyses showed that the prevalence of CD in children with DS was slightly higher than in mixed samples from both children and adults, and the between-study heterogeneity reduced in studies of mixed samples, but not in children group. In addition, the levels of between-study heterogeneity were reduced in studies from America, whereas significant heterogeneity was still found for studies from Europe. However, another explanation of the lower heterogeneity found in these sub-groups is the lower power that the test for heterogeneity has in meta-analysis with smaller number of studies, especially that only six studies from America were included in this meta-analysis. Furthermore, meta-regression analyses indicated that proportion of antibody-positive individuals that underwent small intestine biopsy had moderating effect on the outcome of the meta-analysis, with percentage of biopsied samples positively correlated with effective size. The result suggested that the actual prevalence of CD in patients with DS is likely to be higher than the pooled prevalence found in this meta-analysis, considering it is reasonable that uniformly perform small intestine biopsies in DS patients with positive serologies would automatically increase the prevalence of biopsy-verified CD.

In addition to the lack of uniformly performing small intestine biopsies in serology positive DS patients, one limitation of this meta-analysis is that serologic screening in some studies used older antigliadin antibody (Table 1), and it is now generally considered that antigliadin antibody has a low predictive value for CD, therefore the use of antigliadin antibody for serologic screening in several studies included in this meta-analysis may have led to lower prevalence of CD in patients with DS. In addition, the discovery of tissue transglutaminase as the autoantigen of CD was around year 2000, and it is unclear whether research on screening of CD with tissue transglutaminase antibodies after year 2000 affected the observed prevalence of CD in DS. Another limitation of this study is that we only included English papers for analysis, although we were aware of several non-English papers in the literature, as we were unable to control data and assess study quality for non- English papers. However, the exclusion of the non-English papers is unlikely to affect the high prevalence of CD in DS found in this study, due to the limited number of non-English papers. Indeed, a Portuguese-language article with English-language abstract reported a 5.6 % prevalence of biopsied confirmed CD in DS patients [51], and this is consistent with the pooled prevalence of CD in DS patients found in this meta-analysis. Furthermore, we only included papers from PubMed, Web of Science and CNKI, therefore we can not rule out that potential papers from other databases or unpublished data influence our results. However, as part of this study we performed a publication bias analysis, and it suggested a higher prevalence of CD in DS patients after bias has been taken into account, demonstrating the robustness of our conclusion in this meta-analysis. Finally, we have only identified four studies from continents other than America and Europe, and the pooled prevalence of CD in DS patients is 4.5 % for the four studies, with 6 % in India [19], 3.4 % in Austria [25], 2 % in Saudi Arabia [18] and 3.8 % in Israel [36]. The limited number of studies with small sample sizes in regions other than America and Europe require future studies to verify the findings, and thereby providing better treatment and management of CD in patients with DS globally.

**Table 1:** Papers included in the meta-analysis on celiac disease prevalence in Down syndrome patients

Study/Year	Country	Sample Size	Gender (% Male)	Mean Age (Year)	CD Patients	Antibody	Biopsied (%)
Alanay et al. 2005	Turkey	100	56	6.01 (2-14)	0	EMA	0
Bhat et al. 2013	India	100	56	(2-18)	6	EMA/TTG	85.7
Bonamico et al. 2001	Italy	1202.00	50.7	(1.25-46)	55	AGA/EMA	84.600
Carlsson et al. 1998	Sweden	43.00	48.8	5.8 (1-14)	8	AGA/EMA	83.3
Carnicer et al. 2001	Spain	284.00	NA	(1-25)	18	AGA/EMA	100
Castro et al. 1993	Italy	155.00	64.5	6.25 (0.5-16.42)	7	AGA	51.2
Cataldo et al. 2005	Italy	303.00	NA	> 1	10	AGA/EMA/TTG	55.6
Cerqueira et al. 2010	Portugal	98.00	59.2	(1-45)	9	EMA/TTG	100
Csizmadia et al. 2000	Netherlands	137.00	50	5.3 (1-17.6)	11	EMA	100
Failla et al. 1996	Italy	57.00	50.9	14.9 (1.7-49)	7	AGA	100
Gale et al. 1997	Austrilia	59.00	50.9	37 (25-62)	2	AGA/EMA	100
George et al. 1996	Netherlands	115.00	58.8	5.8	8	AGA/EMA	79.1
Gomes et al. 2015	Brazil	77.00	NA	5.97	10	EMA/TTG	71.4
Hansson et al. 2005	Sweden	72.00	54.2	(1-18)	4	EMA/TTG	84.6
Jansson et al. 1995	Sweden	65.00	46.2	(0-18)	11	AGA/EMA	86.4
Kanavin et al. 1988	Norway	38.00	47.9	(16-62)	2	TMA/TGA	100
Kolek et al. 2003	Czech	25.00	56	(3.1-18)	3	EMA	75
Mackey et al. 2001	US	93.00	61.3	5.6 (1-22)	4	EMA	80
Pavlovic et al. 2010	Serbia	82.00	56.1	4.6 (0.67-8.6)	0	TTG	100
Pueschel et al. 1999	US	105.00	57.1	(2-28)	4	AGA/EMA	80
Rumbo et al. 2002	Argentina	56.00	60.7	4.5 (1-17)	2	AGA/EMA/TTG	100%
Saadab et al. 2012	Saudi Arabia	51.00	74.4	4.69 (0.57-16.64)	1	TTG	100%
Sa´nchez-Albisua et al. 2002	Germany	76.00	48.7	7.2 (1.4-42)	2	AGA/EMA	33.3
Sciberras et al. 2004	Malta	122.00	47	(1-30)	2	AGA/EMA	37.5
Shamaly et al. 2007	Israel	52.00	63.5	11.5^*^	2	AGA/EMA/TTG	83.3
Storm et al. 1990	Germany	78.00	NA	(1-19)	2	AGA	66.7
Szaflarska-Popławska et al. 2016	Poland	301.00	54.5	(1-34)	17	TTG/DGP	74.2
Uibo et al. 2006	Estonia	134.00	45.5	11 (0.5-45)	4	AGA/EMA/TTG	100
Wouters et al. 2009	Netherlands	155.00	62.6	7.4 (0.17-19)	8	EMA/TTG	87.5
Zachor et al. 2000	US	75.00	60.5	(0.83-30)	4	AGA/EMA	50
Zubillage et al. 1993	US	73.00	64.3	6.1 (1-14)	3	AGA/EMA	88.9

Abbreviation: CD, Celiac disease; AGA, Antigliadin antibody; EMA, Antiendomysium antibody; TTG, Tissue transglutaminase antibody; DGP, Deamidated gliadin peptide; “^*^”, median age.

In conclusion, individuals with DS are at very high risk of CD, and more than one in twenty patients (children) with DS have CD, at least in Europe and America. The high prevalence of CD in DS patients found in this meta-analysis should motivate screening for CD in patients with DS.

## MATERIALS AND METHODS

### Search strategy and study selection

We searched articles in the databases of PubMed, Web of Science and China National Knowledge Infrastructure (CNKI) for (Celiac disease or Coeliac disease) and Down syndrome published until August 2017. The search was conducted by two independent researchers. Original clinical studies that reported data on prevalence of CD in DS patients were included. Excluded criteria were: (1) no small intestinal biopsy data; (2) samples were overlapping with other studies; (3) studies were case reports; (4) full-text was not English-language publications

### Data extraction

We retrieved the data by two independent investigators, data on sample size and biopsy-confirmed CD patients we extracted as primary outcomes for meta-analysis. Data on age, gender (proportion of male), country, publication year, and proportion of antibody-positive individuals that underwent small intestine biopsy were also extracted for potential moderator analyses. It should be noted that all the studies included in this meta-analysis were cross-sectional studies, and the diagnosis of CD was made by villous atrophy in the small intestine.

Although we define children are individuals aged between 0-18 year in this study as classified by Pubmed, we can not rule out that some studies included individuals aged between 19-21 years as children, given other definitions of age-groups occur. The study quality was not graded in this meta-analysis, but we chose to discuss several aspects of study qualities in the discussion.

### Statistical analysis

We used Comprehensive Meta-Analysis Version 2 software (Biostat Inc., Englewood, NJ, USA) to perform all the statistical analyses. A random-effects model was chosen when conducting the meta-analysis of the prevalence of CD in DS patients. Effective size and 95 % CIs were calculated. Between-study heterogeneity was assessed by I squared (I^2^), and I^2^ of 0.25, 0.50 and 0.75 indicate small, moderate and high levels of heterogeneity, respectively [52]. To investigate heterogeneity, we performed sub-group analyses based on age group (children and adult) and continent (Europe, America and others). In addition, meta-regression analyses were carried out to test whether the continuous variables including sample size, publication year, gender (proportion of male) and proportion of antibody-positive individuals that underwent small intestine biopsy had moderating effects on the outcome the meta-analysis.

We also used sensitivity analysis by removing one study at a time to assess whether a single study influenced the outcome of the meta-analysis. In addition, publication bias of studies included in this meta-analysis was analyzed by funnel plot. In case of publication bias, we performed the trim-and-fill procedure to estimate an effective size after bias has been taken into account [53].

P < 0.05 was considered statistical significant in this study.

## References

[1] Szaflarska-Poplawska A, Soroczynska-Wrzyszcz A, Barg E, Jozefczuk J, Korczowski B, Grzybowska-Chlebowczyk U, Wiecek S, Cukrowska B (2016). Assessment of coeliac disease prevalence in patients with Down syndrome in Poland - a multi-centre study. Prz Gastroenterol.

[2] Green PH, Jabri B (2003). Coeliac disease. Lancet.

[3] Fasano A, Catassi C (2012). Clinical practice. Celiac disease. N Engl J Med.

[4] Singh P, Arora S, Singh A, Strand TA, Makharia GK (2016). Prevalence of celiac disease in Asia: A systematic review and meta-analysis. J Gastroenterol Hepatol.

[5] Frohlich-Reiterer EE, Hofer S, Kaspers S, Herbst A, Kordonouri O, Schwarz HP, Schober E, Grabert M, Holl RW, DPV-Wiss Study Group (2008). Screening frequency for celiac disease and autoimmune thyroiditis in children and adolescents with type 1 diabetes mellitus--data from a German/Austrian multicentre survey. Pediatr Diabetes.

[6] Uibo O, Heilman K, Rago T, Shor R, Paal M, Metskula K, Tillmann V, Uibo R (2010). Symptomless celiac disease in type 1 diabetes: 12-year experience in Estonia. Pediatr Int.

[7] Mainardi E, Montanelli A, Dotti M, Nano R, Moscato G (2002). Thyroid-related autoantibodies and celiac disease: a role for a gluten-free diet?. J Clin Gastroenterol.

[8] Meloni GF, Tomasi PA, Bertoncelli A, Fanciulli G, Delitala G, Meloni T (2001). Prevalence of silent celiac disease in patients with autoimmune thyroiditis from Northern Sardinia. J Endocrinol Invest.

[9] Villalta D, Girolami D, Bidoli E, Bizzaro N, Tampoia M, Liguori M, Pradella M, Tonutti E, Tozzoli R (2005). High prevalence of celiac disease in autoimmune hepatitis detected by anti-tissue tranglutaminase autoantibodies. J Clin Lab Anal.

[10] Costa Gomes R, Cerqueira Maia J, Fernando Arrais R, Andre Nunes Jatoba C, Auxiliadora Carvalho Rocha M, Edinilma Felinto Brito M, Laissa Oliveira Nazion A, Marques Maranhao C, De Sousa Maranhao H (2016). The celiac iceberg: from the clinical spectrum to serology and histopathology in children and adolescents with type 1 diabetes mellitus and Down syndrome. Scand J Gastroenterol.

[11] Elfstrom P, Sundstrom J, Ludvigsson JF (2014). Systematic review with meta-analysis: associations between coeliac disease and type 1 diabetes. Aliment Pharmacol Ther.

[12] Roy A, Laszkowska M, Sundstrom J, Lebwohl B, Green PH, Kampe O, Ludvigsson JF (2016). Prevalence of Celiac Disease in Patients with Autoimmune Thyroid Disease: A Meta-Analysis. Thyroid.

[13] Alanay Y, Boduroglu K, Tuncbilek E (2005). Celiac disease screening in 100 Turkish children with Down syndrome. Turk J Pediatr.

[14] Carlsson A, Axelsson I, Borulf S, Bredberg A, Forslund M, Lindberg B, Sjoberg K, Ivarsson SA (1998). Prevalence of IgA-antigliadin antibodies and IgA-antiendomysium antibodies related to celiac disease in children with Down syndrome. Pediatrics.

[15] Carnicer J, Farre C, Varea V, Vilar P, Moreno J, Artigas J (2001). Prevalence of coeliac disease in Down's syndrome. Eur J Gastroenterol Hepatol.

[16] Cataldo F, Scola L, Piccione M, Giuffre M, Crivello A, Forte GI, Lio D, Corsello G (2005). Evaluation of cytokine polymorphisms (TNFalpha, IFNgamma and IL-10) in Down patients with coeliac disease. Dig Liver Dis.

[17] Hansson T, Dahlbom I, Rogberg S, Nyberg BI, Dahlstrom J, Anneren G, Klareskog L, Dannaeus A (2005). Antitissue transglutaminase and antithyroid autoantibodies in children with Down syndrome and celiac disease. J Pediatr Gastroenterol Nutr.

[18] Saadah OI, Al-Aama JY, Alaifan MA, Bin Talib YY, Al-Mughales JA (2012). Prevalence of celiac disease in children with Down syndrome screened by anti-tissue transglutaminase antibodies. Saudi Med J.

[19] Bhat AS, Chaturvedi MK, Saini S, Bhatnagar S, Gupta N, Sapra S, Gupta SD, Kabra M (2013). Prevalence of celiac disease in Indian children with Down syndrome and its clinical and laboratory predictors. Indian J Pediatr.

[20] Bonamico M, Mariani P, Danesi HM, Crisogianni M, Failla P, Gemme G, Quartino AR, Giannotti A, Castro M, Balli F, Lecora M, Andria G, Guariso G (2001). Prevalence and clinical picture of celiac disease in italian down syndrome patients: a multicenter study. J Pediatr Gastroenterol Nutr.

[21] Castro M, Crino A, Papadatou B, Purpura M, Giannotti A, Ferretti F, Colistro F, Mottola L, Digilio MC, Lucidi V (1993). Down's syndrome and celiac disease: the prevalence of high IgA-antigliadin antibodies and HLA-DR and DQ antigens in trisomy 21. J Pediatr Gastroenterol Nutr.

[22] Cerqueira RM, Rocha CM, Fernandes CD, Correia MR (2010). Celiac disease in Portuguese children and adults with Down syndrome. Eur J Gastroenterol Hepatol.

[23] Csizmadia CG, Mearin ML, Oren A, Kromhout A, Crusius JB, von Blomberg BM, Pena AS, Wiggers MN, Vandenbroucke JP (2000). Accuracy and cost-effectiveness of a new strategy to screen for celiac disease in children with Down syndrome. J Pediatr.

[24] Failla P, Ruberto C, Pagano MC, Lombardo M, Bottaro G, Perichon B, Krishnamoorthy R, Romano C, Ragusa A (1996). Celiac disease in Down's syndrome with HLA serological and molecular studies. J Pediatr Gastroenterol Nutr.

[25] Gale L, Wimalaratna H, Brotodiharjo A, Duggan JM (1997). Down's syndrome is strongly associated with coeliac disease. Gut.

[26] George EK, Mearin ML, Bouquet J, von Blomberg BM, Stapel SO, van Elburg RM, de Graaf EA (1996). High frequency of celiac disease in Down syndrome. J Pediatr.

[27] Jansson U, Johansson C (1995). Down syndrome and celiac disease. J Pediatr Gastroenterol Nutr.

[28] Kanavin O, Scott H, Fausa O, Ek J, Gaarder PI, Brandtzaeg P (1988). Immunological studies of patients with Down's syndrome. Measurements of autoantibodies and serum antibodies to dietary antigens in relation to zinc levels. Acta Med Scand.

[29] Kolek A, Vospelova J, Hermanova Z, Santava A, Tichy M (2003). Occurrence of coeliac disease in children with Down's syndrome in north Moravia, Czech Republic. Eur J Pediatr.

[30] Mackey J, Treem WR, Worley G, Boney A, Hart P, Kishnani PS (2001). Frequency of celiac disease in individuals with Down syndrome in the United States. Clin Pediatr (Phila).

[31] Pavlovic M, Radlovic N, Lekovic Z, Stojsic Z, Puleva K, Berenji K (2010). When to screen children with Down syndrome for celiac disease?. J Trop Pediatr.

[32] Pueschel SM, Romano C, Failla P, Barone C, Pettinato R, Castellano Chiodo A, Plumari DL (1999). A prevalence study of celiac disease in persons with Down syndrome residing in the United States of America. Acta Paediatr.

[33] Rumbo M, Chirdo FG, Ben R, Saldungaray I, Villalobos R (2002). Evaluation of coeliac disease serological markers in Down syndrome patients. Dig Liver Dis.

[34] Sanchez-Albisua I, Storm W, Wascher I, Stern M (2002). How frequent is coeliac disease in Down syndrome?. Eur J Pediatr.

[35] Sciberras C, Vella C, Grech V (2004). The prevalence of coeliac disease in Down's syndrome in Malta. Ann Trop Paediatr.

[36] Shamaly H, Hartman C, Pollack S, Hujerat M, Katz R, Gideoni O, Shamir R (2007). Tissue transglutaminase antibodies are a useful serological marker for the diagnosis of celiac disease in patients with Down syndrome. J Pediatr Gastroenterol Nutr.

[37] Storm W (1990). Prevalence and diagnostic significance of gliadin antibodies in children with Down syndrome. Eur J Pediatr.

[38] Uibo O, Teesalu K, Metskula K, Reimand T, Saat R, Sillat T, Reimand K, Talvik T, Uibo R (2006). Screening for celiac disease in Down's syndrome patients revealed cases of subtotal villous atrophy without typical for celiac disease HLA-DQ and tissue transglutaminase antibodies. World J Gastroenterol.

[39] Wouters J, Weijerman ME, van Furth AM, Schreurs MW, Crusius JB, von Blomberg BM, de Baaij LR, Broers CJ, Gemke RJ (2009). Prospective human leukocyte antigen, endomysium immunoglobulin A antibodies, and transglutaminase antibodies testing for celiac disease in children with Down syndrome. J Pediatr.

[40] Zachor DA, Mroczek-Musulman E, Brown P (2000). Prevalence of celiac disease in Down syndrome in the United States. J Pediatr Gastroenterol Nutr.

[41] Zubillaga P, Vitoria JC, Arrieta A, Echaniz P, Garcia-Masdevall MD (1993). Down's syndrome and celiac disease. J Pediatr Gastroenterol Nutr.

[42] Marild K, Stephansson O, Grahnquist L, Cnattingius S, Soderman G, Ludvigsson JF (2013). Down syndrome is associated with elevated risk of celiac disease: a nationwide case-control study. J Pediatr.

[43] Dias JA, Walker-Smith J (1990). Down's syndrome and coeliac disease. J Pediatr Gastroenterol Nutr.

[44] Hill ID, Dirks MH, Liptak GS, Colletti RB, Fasano A, Guandalini S, Hoffenberg EJ, Horvath K, Murray JA, Pivor M, Seidman EG, North American Society for Pediatric Gastroenterology, Hepatology and Nutrition (2005). Guideline for the diagnosis and treatment of celiac disease in children: recommendations of the North American Society for Pediatric Gastroenterology, Hepatology and Nutrition. J Pediatr Gastroenterol Nutr.

[45] Bull MJ, Committee on Genetics (2011). Health supervision for children with Down syndrome. Pediatrics.

[46] Sollid LM, Thorsby E (1993). HLA susceptibility genes in celiac disease: genetic mapping and role in pathogenesis. Gastroenterology.

[47] Sollid LM, Lie BA (2005). Celiac disease genetics: current concepts and practical applications. Clin Gastroenterol Hepatol.

[48] Broers CJ, Gemke RJ, Weijerman ME, van der Sluijs KF, van Furth AM (2012). Increased pro-inflammatory cytokine production in Down Syndrome children upon stimulation with live influenza A virus. J Clin Immunol.

[49] Carta MG, Serra P, Ghiani A, Manca E, Hardoy MC, Del Giacco GS, Diaz G, Carpiniello B, Manconi PE (2002). Chemokines and pro-inflammatory cytokines in Down's syndrome: an early marker for Alzheimer-type dementia?. Psychother Psychosom.

[50] Zaki ME, El-Bassyouni HT, Tosson AM, Youness E, Hussein J (2017). Coenzyme Q10 and pro-inflammatory markers in children with Down syndrome: clinical and biochemical aspects. J Pediatr (Rio J).

[51] Nisihara RM, Kotze LM, Utiyama SR, Oliveira NP, Fiedler PT, Messias-Reason IT (2005). Celiac disease in children and adolescents with Down syndrome. J Pediatr (Rio J).

[52] Qin XY, Cao C, Cawley NX, Liu TT, Yuan J, Loh YP, Cheng Y (2017). Decreased peripheral brain-derived neurotrophic factor levels in Alzheimer's disease: a meta-analysis study (N=7277). Molecular Psychiatry.

[53] Qin XY, Wu HT, Cao C, Loh YP, Cheng Y (2017). A meta-analysis of peripheral blood nerve growth factor levels in patients with schizophrenia. Molecular Psychiatry.

